# Glycocalyx strengthens endothelial barrier function and protects from angioedema inducing compounds

**DOI:** 10.3389/fimmu.2026.1758997

**Published:** 2026-03-12

**Authors:** Anna Reich, Emily Lehnert, Raphael Möhrle, Angelina Gierke, Cornelia Brunner, Thomas K. Hoffmann, Jens Greve, Janina Hahn, Robin Lochbaum

**Affiliations:** 1 Department of Otorhinolaryngology, Head and Neck Surgery, Ulm University Medical Center, Ulm, Germany; 2 Core Facility Immune Monitoring, Ulm University Medical Faculty, Ulm, Germany

**Keywords:** biomarker, bradykinin, endothelial barrier, histamine, serotonin

## Abstract

**Introduction:**

Hereditary angioedema (HAE) and mast cell-mediated angioedema are characterized by episodic swelling. Yet, atypical variants, such as HAE with normal C1 inhibitor, remain poorly understood and diagnostically challenging. Although advances in diagnosis and therapy have improved patient outcomes, investigating the role of the endothelial glycocalyx in regulating vascular permeability may uncover novel biomarkers and enhance both diagnostic and prognostic strategies.

**Materials and methods:**

Human umbilical vein endothelial cells (HUVEC) were treated with enzymes for glycocalyx degradation. Glycocalyx quantification was performed using wheat germ agglutinin (WGA) assays, enzyme-linked immunosorbent assays, and real time-polymerase chain reaction. Endothelial barrier function was evaluated through measurements of transendothelial electrical resistance (TEER), and permeability for dextrans. Water flux across the endothelium was determined using the D_2_O dilution method.

**Results:**

HUVEC expressed a functional glycocalyx. Enzymatic glycocalyx degradation reduced barrier integrity. Bradykinin and serotonin did not affect glycocalyx integrity, while histamine reduced WGA binding. Bradykinin and histamine also decreased hyaluronidase activity. Barrier function analysis showed that glycocalyx degradation and compound exposure led to increased water permeability, with no additive effects on TEER and permeability.

**Discussion:**

Glycocalyx degradation increased endothelial permeability, with possible implication for edema development. Bradykinin and serotonin did not affect glycocalyx composition, but histamine reduced its integrity. Glycocalyx degradation led to a further increase in mediator-induced water permeability. This suggests a protective role of the glycocalyx endothelial dysfunction. This highlights its potential as a biomarker for angioedema.

## Introduction

Angioedema is characterized by transient, localized swelling of the deeper layers of the skin and mucosa resulting from increased vascular permeability and plasma extravasation. The clinical manifestation of angioedema can rapidly become life-threatening if edema formation affects the airways. Therefore, understanding the pathogenesis of the disease is crucial for early identification of at-risk patient groups and for educating them on potential triggers and appropriate treatment in the event of symptoms.

According to current classifications, angioedema can be broadly divided into bradykinin-mediated, mast cell–mediated, and certain drug-induced or endothelium driven forms with heterogeneous mechanisms (1). Mast cell-mediated angioedema arises, among other factors, from the release of vasoactive compounds like histamine or serotonin and occurs significantly more frequently than bradykinin-mediated angioedema (2). While mast cell-mediated angioedema is typically accompanied by pruritus and wheal formation, these symptoms are absent in bradykinin-mediated angioedema (3). Bradykinin-mediated angioedema comprises different subtypes based on its pathogenesis. Hereditary angioedema (HAE) is most commonly caused by mutations in the SERPING1 gene encoding C1 inhibitor (C1-INH). Reduced functional C1-INH permits excessive activation of the plasma kallikrein-kinin-system, including factor XII and plasma kallikrein, resulting in cleavage of high-molecular-weight kininogen and increased bradykinin release (4, 5). In addition to C1-INH deficiency, several forms of HAE with normal C1-INH have been identified that are associated with mutations in genes such as *F12*, *PLG*, *ANGPT1*, and more recently *HS3ST6*, encoding heparan sulfate 3-O-sulfotransferase 6. Elevated bradykinin enhances endothelial permeability via B2 receptor activation. In contrast, drug-induced angioedema results from impaired bradykinin degradation, primarily due to inhibition of angiotensin-converting enzyme (ACE) (6–8). Although effective therapies exist for various forms of angioedema, clinical management remains challenging due to difficulties in diagnosis, classification of the underlying mechanism, assessment of disease burden, and prediction of treatment response. These challenges emphasize the need for reliable and easily accessible biomarkers to support accurate diagnosis, determine the pathophysiological subtype, evaluate disease severity, and guide individualized treatment decisions in both acute and chronic care settings (9).

Angioedema results from increased endothelial permeability, leading to enhanced fluid extravasation into the interstitial tissue. The capillary wall comprises endothelial cells and a basement membrane, which, through cell-cell junctions and the glycocalyx, maintain a spatial separation between blood and interstitial fluid (10). The endothelial barrier function is predominantly mediated by tight junctions and adherens junctions, which overlap within the lateral intercellular space of endothelial cells (11). In addition to them, the glycocalyx is a critical component of the endothelial barrier. Located on the apical surface of endothelial cells, it forms a polysaccharide-rich meshwork (12). Curry et al. proposed a two-layered glycocalyx structure: the inner layer is a dense network of membrane-bound glycoproteins, while the outer layer, extending up to one micrometer into the lumen, contains glycosaminoglycans (GAGs), proteoglycans, and plasma proteins like albumin and antithrombin (13). The most relevant proteoglycans of the glycocalyx include glypicans and syndecans which are connected to the plasma membrane (14). Other proteoglycans, such as perlecan, mimecan, biglycan, and versican, are rather secreted by endothelial cells than remaining membrane bound. The negatively charged GAGs, including heparan sulfate, and chondroitin sulfate, are covalently attached to proteoglycans, with heparan sulfate representing the majority of total GAGs, followed by chondroitin sulfate and hyaluronic acid (15, 16). Glycocalyx thickness in Human umbilical vein endothelial cells (HUVEC) ranges from 30 nm (electron microscopy) to 2.5 µm (confocal laser microscopy), with significant variation depending on environmental conditions and measurement methods (17, 18). For instance, at the carotid bifurcation, glycocalyx thickness is significantly reduced due to shear stress compared to segments of the carotid artery with uniform blood flow (19).

The glycocalyx has multiple functions, with endothelial permeability regulation being particularly relevant to the pathophysiology of angioedema. Adamson et al. demonstrated that an oncotic pressure gradient forms across the glycocalyx toward the capillary lumen due to a protein-depleted subglycocalyx space apical to tight junctions (20). The negatively charged sulfate groups of GAGs further contribute to glycocalyx semipermeability by attracting cations and binding albumin at physiological pH, thereby maintaining the oncotic gradient (18). The glycocalyx permits only smaller plasma proteins, such as albumin, to pass through its seven-nanometer pores, preventing interactions with blood components like platelets and leukocytes (21). When the glycocalyx is degraded, platelet adhesion becomes possible, potentially leading to microthrombi and severe disseminated intravascular coagulation (22, 23). Additionally, the glycocalyx functions as a mechanoreceptor by detecting shear forces via glypican-1 and its associated heparan sulfates (14, 24).

Glycocalyx degradation is implicated in various systemic diseases. Elevated plasma levels of syndecan-1 correlate with sepsis severity and coagulation disturbances (25). The measurement of GAG fragments in urine samples from patients with septic shock or acute respiratory distress syndrome can aid in predicting renal failure within 72 hours and overall mortality (26). Atherogenesis is also associated with glycocalyx impairment, particularly in regions of turbulent, non-uniform blood flow (27). While glycocalyx integrity likely plays a role in all vascular diseases, its precise involvement in angioedema pathogenesis remains unknown, but could also serve as a potential biomarker for diagnostics and prognostic assessment. The objective of this study was to investigate how glycocalyx degradation and exposure to vasoactive mediators affect endothelial barrier function, in order to clarify its role in angioedema pathogenesis and to evaluate its potential as a diagnostic and prognostic biomarker.

## Materials and methods

### Cell culture

The experiments were conducted on HUVEC obtained from PromoCell GmbH, Heidelberg. The HUVEC were cultured in a specialized cell culture medium from the Endothelial Cell Growth Medium 2 Kit (*PromoCell GmbH, Heidelberg*), supplemented with ascorbic acid (1 μg/ml), fetal calf serum (FCS, 0.02 ml/ml), heparin (22.5 μg/ml), human basic fibroblast growth factor (FGF, 10 ng/ml), human epidermal growth factor (EGF, 5 ng/ml), human insulin-like growth factor (IGF, 20 ng/ml), human vascular endothelial growth factor (VEGF, 0.5 ng/ml), hydrocortisone (0.2 μg/ml), and 2% penicillin/streptomycin (*Pan-Biotech, Aidenbach*). The cells were maintained in an incubator at 17% O2, 5% CO2, and 37 °C. The culture medium was changed twice a week. Once the cells reached 80-90% confluency, they were subcultured into new cell culture flasks or used for experiments. The splitting reagents Hepes BSS, Trypsin/EDTA, and Trypsin Neutralizing Solution (all from *PromoCell GmbH, Heidelberg*) were utilized for subculturing. The cell culture plates and filter inserts (*Sarstedt AG & Co. KG, Nümbrecht*) used in the experiments were pre-coated with a 50 μg/ml collagen I solution (*Corning GmbH, Kaiserslautern*). The experiments were consistently performed on the fourth day after seeding and with at least two passages. For glycocalyx degradation, the following enzymes were used: Neuraminidase from *Clostridium perfringens* (0.12 U/ml), Chondroitinase ABC from *Proteus vulgaris* (0.012 U/ml), Heparanase-1 pre-activated human (0.04 μg/ml), and Hyaluronidase from bovine testes (1.2 mg/ml) (all from *Sigma-Aldrich Chemie GmbH, Steinheim*). The cells were incubated for six hours with each enzyme individually or with all enzymes combined. Additionally, for some experiments, the following modulators were applied: Bradykinin (100 µM), histamine (0,1 µM), and serotonin (100 µM, all from *Sigma-Aldrich Chemie GmbH, Steinheim*). When combining enzymes with bradykinin, histamine, and serotonin, cells were first incubated with enzymes for six hours, followed by the addition of the respective modulator and further incubation for four hours before the experiment. Bright-field images were acquired using an Olympus CK30 microscope (*Carl Zeiss AG, Oberkochen*).

### Wheat germ agglutinin assay

To visualize and quantify the glycocalyx, a Wheat Germ Agglutinin (WGA) assay was performed. WGA stock solution (1.0 mg/ml) and WGA labeling solution (5.0 μg/ml) were prepared according to the manufacturer’s protocol and stored at -20 °C under light protection (*Thermo Fisher Scientific, Karlsruhe*). HUVEC were cultured in 96-well plates (10,000 cells/well, *Sarstedt AG & Co. KG, Nümbrecht*) and incubated with the respective enzymes and modulators. After washing with PBS (*Pan-Biotech, Aidenbach*), the cells were incubated with the WGA labeling solution at 37 °C for 10 minutes. Following two PBS washes, Hank’s Balanced Salt Solution (*Thermo Fisher Scientific, Karlsruhe*) was added, and fluorescence intensity was measured using a TECAN reader with the i-control software (*Tecan Group AG, Männedorf, Switzerland*). The excitation and emission wavelengths were set to 480 nm and 516 nm, respectively. The number of flashes was set to 25, and the integration time was 20 μs.

### Enzymatic activities of heparanase and hyaluronidase

Initially, HUVEC were cultured in 24-well plates for four days. The enzymatic activities of heparanase and hyaluronidase were determined using the respective activity kits from Amsbio (*Amsbio Europe B.V., Alkmaar*) and performed according to the manufacturer’s protocol. In this assay, the cell supernatant containing the target enzymes was applied to 96-well plates pre-coated with the corresponding substrates. Subsequently, optical density was measured at 450 nm using streptavidin–horseradish peroxidase, enabling quantification of the remaining substrate and thus determination of enzymatic activity. A decrease in optical density is directly proportional to the enzymatic activity present in the supernatant. Kit-provided enzymes served as positive controls, while negative controls consisted of wells receiving reaction buffer only.

### Resazurin viability assay

Resazurin, a non-fluorescent blue indicator dye, undergoes mitochondrial reduction exclusively in metabolically active cells, yielding the fluorescent product resorufin. The intensity of the generated fluorescence is proportional to the number of viable cells and can therefore be used as a quantitative measure of cellular proliferation or metabolic activity. For all experiments, the Resazurin Assay Kit (*Abcam, Cambridge*) was applied in accordance with the manufacturer’s protocol: 10,000 cells were seeded into dark-walled 96-well plates in 100 µl culture medium. For each experimental condition, wells containing cells without dye were included to determine background signals. At the end of the treatment period, 100 µl of resazurin solution was added to each well and incubated for 4 h at 37 °C. Fluorescence was then measured at 590 nm (excitation 550 nm). Background values from wells without dye were subtracted prior to correction.

### Reverse transcription polymerase chain reaction

Reverse Transcription Polymerase Chain Reaction (RT-PCR) was performed to analyze the gene expression of specific glycocalyx components in HUVEC. RNA isolation was performed according to the RNeasy Mini Kit and RNase-Free DNase Set protocols (both from *Qiagen GmbH, Hilden*). cDNA synthesis followed the protocol of the QuantiTect Reverse Transcription Kit (*Qiagen GmbH, Hilden*) using the peqSTAR 96X Universal Gradient Thermocycler (*VWR International GmbH, Darmstadt*) at 42 °C for 15 minutes and 95 °C for 3 minutes. The obtained cDNA was diluted 1:3 with RNase-free water. Primer sequences for RT-PCR were selected using the NCBI database and synthesized by Biomers (*biomers.net GmbH, Ulm*). Primers were dissolved in nuclease-free water as specified in the datasheet and diluted 1:10. Glyceraldehyde-3-phosphate dehydrogenase (GAPDH) was used as the housekeeping gene. Used primers can be found in the supplements. Biglycan (forward: tcccagacctcaagctcct, reverse: tgggacagaagtcgttgaca), GAPDH (forward: agccacatcgctcagacac; reverse: gcccaatacgaccaaatcc), GPC-1 (forward: cgaggtccgccagatctac, reverse: gacagatccgcaggtgct), GPC-2 (forward: ctgggacacgacctggac, reverse: ccagccatccagtcatctg), HSPG2 (forward: tgagtccttctactggcagc, reverse: gggcctgctgtgtaggagag), SDC-2 (forward: gtggatcctgctcaccttg, reverse: gtctttatcagatgtcagctctgc), SDC-3 (forward: cggaaggaggtgctcgtag, reverse: accaagaaggcagcaaagag), SDC-4 (forward: ggcaggaatctgatgactttg, reverse: ggccgatcatggagtcttc) and Versican (forward: tgggaaaggcaggagtcagg, reverse: gcagcctcctcgaaggtgaa). RT-PCR was conducted following the QuantiNova™ SYBR^®^ Green PCR Kit protocol (*QIAGEN GmbH, Hilden*). PCR cycles were performed in the LightCycler^®^ 96 System (*Roche Deutschland Holding GmbH, Grenzach-Wyhlen*) at 95°C for 2 min, followed by 40 cycles at 95°C for 5 s and 60°C for 10 s.

### Immunocytochemistry

Cells cultured on transwell inserts were subjected to two initial washes with calcium- and magnesium-containing phosphate-buffered saline (PBS; *PAN-Biotech GmbH, Aidenbach*). Fixation was performed by incubating the cells in 4% formaldehyde (*Thermo Fisher Scientific, Karlsruhe*) prepared in PBS for 10–20 minutes. Subsequently, three PBS washes were carried out to remove residual fixative. Permeabilization was achieved by exposing the samples to 0.1% Triton X-100 (*Sigma Aldrich Chemie GmbH, Steinheim*) in PBS for 5 minutes, followed by two sequential 5-minute incubations in 0.5% Triton X-100 supplemented with 2% fetal calf serum (FCS) in PBS. Primary antibodies specific for vascular endothelial cadherin (VE-cadherin, a key component of endothelial adherens junctions; Polyclonal Rabbit IgG, 36-1900, *Thermo Fisher Scientific, Karlsruhe*) and zonula occludens-1 (ZO-1, a tight junction–associated scaffold protein; Rabbit antibody, ab221547, *Abcam, Cambridge*) were prepared at a 1:100 dilution in PBS containing 2% FCS and applied for 1 h at ambient temperature. Excess antibody was removed by three washes in PBS/2% FCS. The secondary antibody (Donkey anti-Rabbit IgG (H+L), Alexa Fluor 488, A-21206, *Thermo Fisher Scientific, Karlsruhe*) was diluted 1:400 in PBS/2% FCS and added to the samples together with Hoechst 33342 (1:50,000 dilution; *Thermo Fisher Scientific, Karlsruhe*) for nuclear labeling. Incubation proceeded for 1 h at room temperature. Following staining, samples underwent three washes with PBS/2% FCS and three final washes in PBS alone. The inserts were transferred to ibiTreat μ-Slide 8-Well chambers (*ibidi GmbH, Martinsried*) for imaging. Fluorescence micrographs were acquired using a KEYENCE BZ-9000 imaging system (*Keyence Deutschland GmbH, Neu-Isenburg*), and data processing was performed in FIJI (ImageJ; *National Institutes of Health, Bethesda*).

### Transendothelial electrical resistance

To measure transendothelial electrical resistance (TEER), HUVEC were cultured as described above on Transwell filter inserts. The experiments were consistently performed on the fourth day after seeding (20,000 cells per filter), and confluence was verified by light microscopy prior to measurement. For TEER acquisition, inserts were transferred to the CellZscope system (*nanoAnalytics GmbH, Münster*). A total of 250 µl medium were added apically and 500 µl basally. TEER was recorded using impedance spectroscopy. This measurement allows assessment of endothelial barrier integrity, as increased permeability corresponds to a decrease in electrical resistance.

### Apparent permeability coefficient (P_app_)

HUVEC were cultured on Transwell filter inserts (20,000 cells per filter) and incubated with enzymes, bradykinin, histamine or serotonin on the fourth day after seeding. One hour before the end of incubation, the basal culture medium was replaced with 500 µl of 0.9% NaCl solution, and fluorescein isothiocyanate-dextrans (FITC-dextran, 70 kDa, *Sigma-Aldrich Chemie GmbH, Steinheim*) were added apically at a 1:100 dilution, followed by incubation at 37 °C for the remaining hour. Subsequently, 50 µl of the basal medium was collected and transferred to a 96-well plate (*Sarstedt AG & Co. KG, Nümbrecht*). A standard dilution series with known dextran concentrations ranging from 0 µmol/l to 200 µmol/l was prepared in the same plate. The fluorescence intensity of FITC-dextrans was measured using the TECAN Infinite M200 plate reader with the i-control software (*Tecan Group AG, Männedorf, Switzerland*). The apparent permeability coefficient was calculated using the following equation: P_app_ = (C × V_0_)/(Δt × A × ΔC_0_) where V_0_ = basal volume (500 µl), Δt = incubation time (3600 s), A = filter area (0.33 cm²), ΔC_0_ = concentration difference between apical and basal compartments (400 µM), and C = dextran concentration in the basal compartment, determined from the dilution series.

### Ion-specific permeability

The permeability of charged ions was investigated using ion substitution based on the method of Alexandre et al. (28). TEER values of cells exposed to sodium chloride (NaCl) were compared to those of cells where NaCl was replaced with either sodium aspartate or chloride-lysine buffer. All buffers were prepared as follows: Ampuwa (100 ml, *Fresenius Kabi, Bad Homburg*), 5 mM potassium chloride (0.0375 g, *Merck KGaA, Darmstadt*), 1 mM potassium phosphate (0.0136 g, *Merck KGaA, Darmstadt*), 10 mM HEPES (0.238 g, *Sigma-Aldrich Chemie GmbH, Steinheim*), 1 mM magnesium sulfate heptahydrate (0.0246 g, *Merck KGaA, Darmstadt*), 2.5 mM calcium chloride dihydrate (0.03675 g, *Merck KGaA, Darmstadt*), and 10 mM glucose (0.180 g, *Merck KGaA, Darmstadt*). The sodium chloride buffer contained an additional 140 mM NaCl (0.812 g, *Sigma-Aldrich Chemie GmbH, Steinheim*), the chloride-lysine buffer contained 140 mM Cl-lysine (2.534 g, *Carl Roth, Karlsruhe*), and the sodium aspartate buffer contained 140 mM Na-aspartate (2.786 g, *Merck KGaA, Darmstadt*). The buffers were adjusted to a pH of 7.4 and an osmolality of 275–300 mosm/kg. TEER measurements were performed with the respective buffers by applying 250 µl apically and 500 µl basally of the same buffer. Each filter was measured with every buffer, with washing and buffer replacement between measurements.

### D_2_O dilution method

Transendothelial water flux was quantified using a D_2_O-based dilution approach. This method represents a direct measurement of transendothelial water permeability - an aspect of particular importance for research on angioedema. It was recently developed and implemented in our laboratory and has since been published (29). After three days of cell culture on Transwell filter inserts, a hydrostatic pressure gradient (1 cm H_2_O for 24 h) was generated by adding 0.9% NaCl to the apical compartment to drive water flux. Following incubation, 25 µl of isotonic D_2_O was introduced into the apical chamber, mixed thoroughly, and the entire apical volume was collected. The relative proportions of H_2_O and D_2_O were subsequently determined by Fourier-transform infrared spectroscopy with the Bruker Alpha 2 spectrometer (*Bruker Optics, Ettlingen*), which exploits the distinct absorption spectra of molecular bonds to quantify both isotopes. Based on an established dilution curve, these measurements allowed calculation of the volume of water transported across the endothelial monolayer. The percentage and absolute water content were calculated according to Müller et al.

### Statistical analysis

Data analysis was performed using Prism 10 (*GraphPad, San Diego, USA*). The statistical tests applied are specified in the text. A p-value of less than 0.05 was considered statistically significant. Significance levels were indicated in the figures as follows: * for p < 0.05, ** for p < 0.01, and *** for p < 0.001.

## Results

### Characterization of endothelial glycocalyx in HUVEC

**Figure 1:**
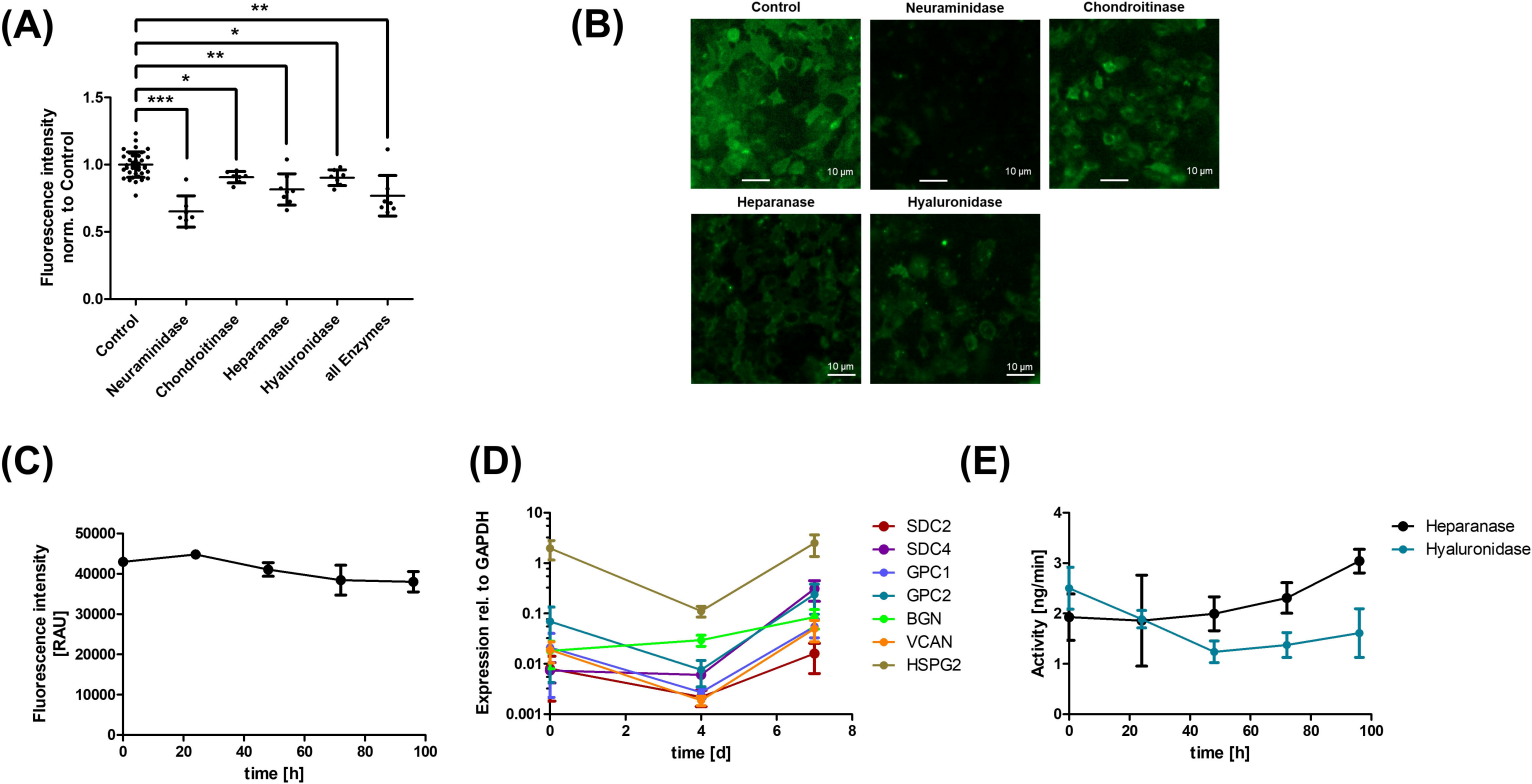
Human umbilical vein endothelial cells (HUVEC) express a glycocalyx. The glycocalyx of HUVEC was degraded using various enzymes as indicated, as well as a combination of all enzymes (all Enzymes), and its thickness was determined by the Wheat Germ Agglutinin (WGA) assay. A reduction in fluorescence signal was observed for all enzymes and their combination [**(A)** representative images are shown in **(B)**]. The thickness of the glycocalyx was then analyzed over the course of cultivation, revealing minimal differences [**(C)** RAU, relative arbitrary unit]. A heterogeneous pattern was observed in the expression dynamics of relevant glycoproteins, determined by RT-PCR [**(D)** SDC, Syndecan; GPC, Glypican; BGN, Biglycan; VCAN, Versican; HSPG2, Perlecan]. Expression was normalized to glyceraldehyde-3-phosphate dehydrogenase (GAPDH). Sheddase activity related to glycocalyx degradation was also investigated, showing an increase in heparanase activity and a decrease in hyaluronidase activity **(E)**. The values in A are shown as scatter plots and the mean value with standard deviation is given. Each data point represents one well. Significance was calculated using the two-tailed Mann-Whitney test, because of multiple testing a Bonferroni correction was additionally performed (*p < 0.05, **p < 0.01, ***p < 0.001). Time course experiments are shown as mean ± standard error of the mean.

HUVEC, a widely used model for studying endothelial barrier function, were investigated for glycocalyx expression and its role in barrier function. Initially, it was investigated whether the cells express a glycocalyx (**Figure 1**). The glycocalyx was enzymatically degraded, and its thickness was measured using WGA, showing a reduction in the WGA signal (A, representative images shown in B). Temporal dynamics of glycocalyx formation were analyzed, revealing a largely constant signal during the 4 days of cultivation (C). RT-PCR was then employed to analyze the relevant expressed glycoproteins (**Supplementary Data 1**). Dynamic analyses of these revealed heterogeneous expression patterns. SDC4 and BGN expressions increased over time, while other genes showed decreased expression after 4 days, with only GPC2 showing a rise above baseline at day 7 (D). Sheddase activity analysis by ELISA revealed that heparanase activity increased, while hyaluronidase activity decreased by day 2 and remained constant (E).

### The glycocalyx in HUVEC promotes their barrier function

**Figure 2:**
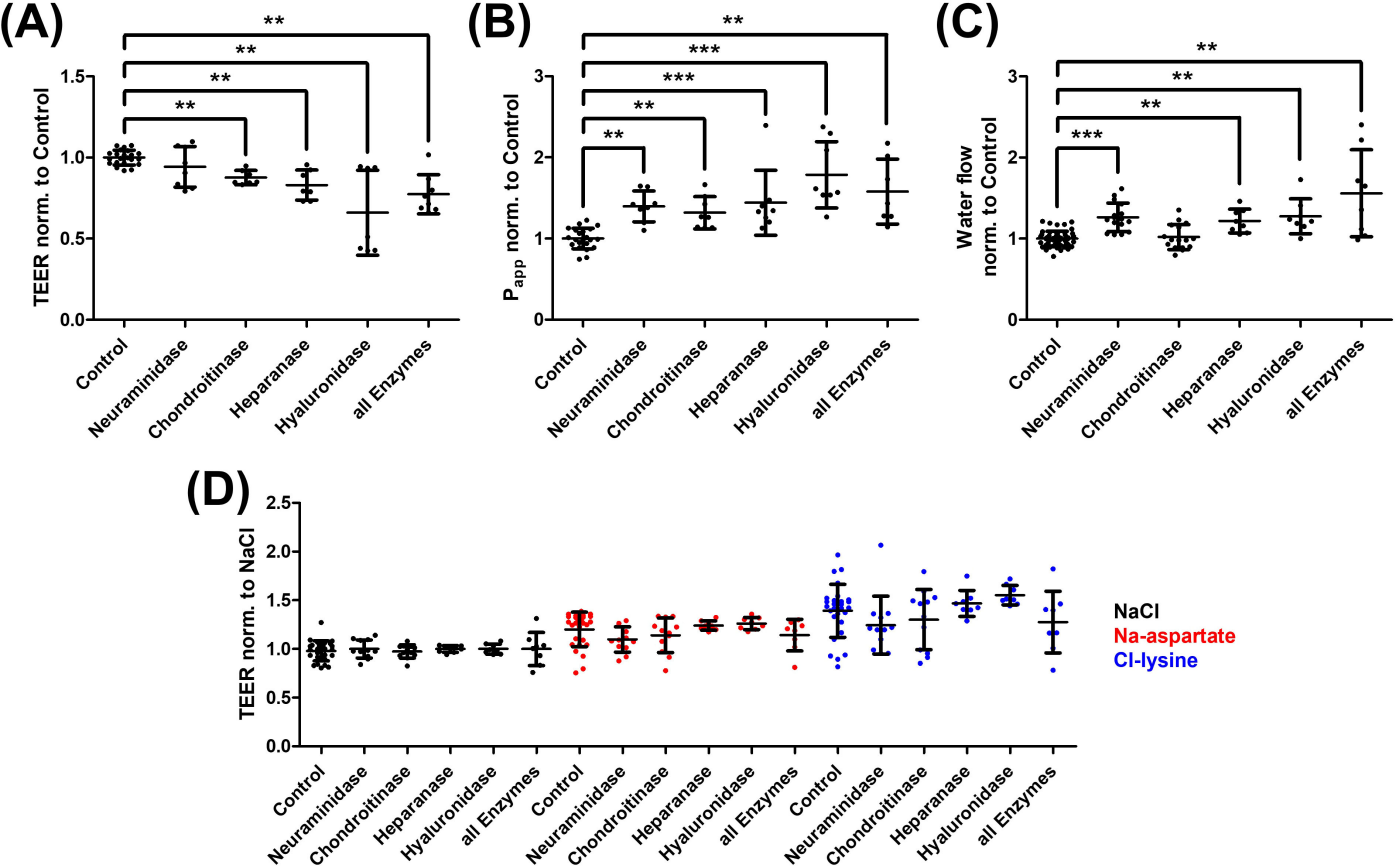
Analysis of the barrier properties regarding the endothelial glycocalyx. The role of the glycocalyx in endothelial barrier function was investigated through enzymatic degradation. Transendothelial electrical resistance (TEER) measurements showed a significant reduction in TEER following glycocalyx degradation, except with neuraminidase **(A)**. Consistently, the apparent permeabi lity coefficient (P_app_) for 70 kDa dextrans was significantly increased with all enzymes and their combination [all Enzymes, **(B)**]. A similar result was observed for transendothelial water flux, measured using the D_2_O-dilution method **(C)**. To test whether the glycocalyx exhibits ion-specific permselective properties, TEER measurements were performed with sodium chloride (NaCl, black), sodium aspartate (Na-aspartate, green), and chloride-lysine (Cl-lysine, purple). As expected, TEER was higher with the latter two buffers, but no significant differences were observed between the control group and the individual enzymes **(D)**. The values are shown as scattered plots, and the mean value with the standard deviation is given. Each data point represents one filter. Significances were calculated using the two-tailed Mann Whitney test, in case of multiple testing a Bonferroni correction was additionally performed (**p < 0.01, ***p < 0.001).

After confirming its expression, the role of the glycocalyx in endothelial barrier function was analyzed (**Figure 2**). Enzymatic degradation was performed again, and treatment with nearly all degrading enzymes resulted in a reduction of TEER (A). Consistently, permeability of 70 kDa dextrans was also significantly increased (B). Except for chondroitinase, all enzymes led to increased water permeability of the endothelium, as measured by the D_2_O-dilution method (C). To assess whether the barrier function of the glycocalyx is ion-specific, TEER measurements were conducted using sodium chloride, sodium aspartate, and chloride-lysine (D). As expected, TEER increased with sodium aspartate and chloride-lysine compared to sodium chloride. However, no significant differences were observed between the glycocalyx degradation and the control group.

### Degradation of the endothelial glycocalyx did not affect cell viability

**Figure 3:**
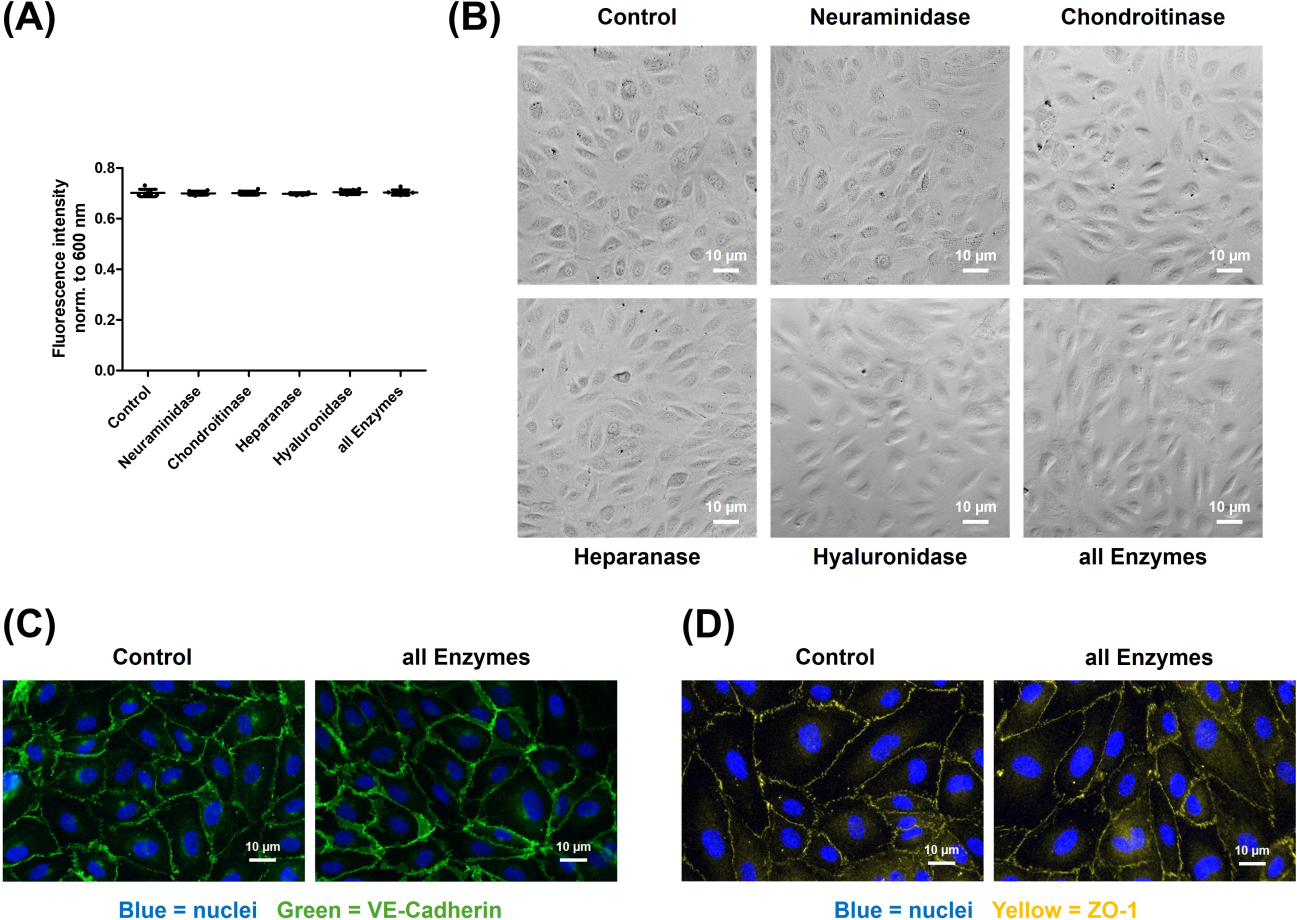
Glycocalyx degradation does not impair endothelial cell viability or junctional organization. Degradation of the endothelial glycocalyx does not affect cell viability. Viability was assessed metabolically using the resazurin assay **(A)** and by bright-field microscopy of the endothelial monolayer **(B)**. No differences were observed between control cells and those subjected to enzymatic glycocalyx degradation. This finding is further supported by immunocytochemical staining of the cell–cell junction proteins VE-cadherin [**(C)** green, nuclei in blue] and ZO-1 [**(D)** yellow, nuclei in blue], which showed no notable changes in localization. The values are shown as scattered plots, and the mean value with the standard deviation is given. Each data point represents one filter. Significances were calculated using the two-tailed Mann Whitney test, in case of multiple testing a Bonferroni correction was additionally performed.

To exclude the possibility that glycocalyx degradation affects cell viability, we assessed metabolic activity and monolayer integrity (**Figure 3**). Degradation of the endothelial glycocalyx did not impair cell viability. Metabolic assessment using the resazurin assay (A) and phase contrast microscopy of the endothelial monolayer (B) revealed no differences between control cells and those subjected to enzymatic glycocalyx degradation. Consistently, immunocytochemical staining of junctional proteins VE-cadherin (C) and ZO-1 (D) showed no notable alterations in localization following glycocalyx degradation.

### Bradykinin, histamine, and serotonin have a divergent effect on the endothelial glycocalyx

**Figure 4:**
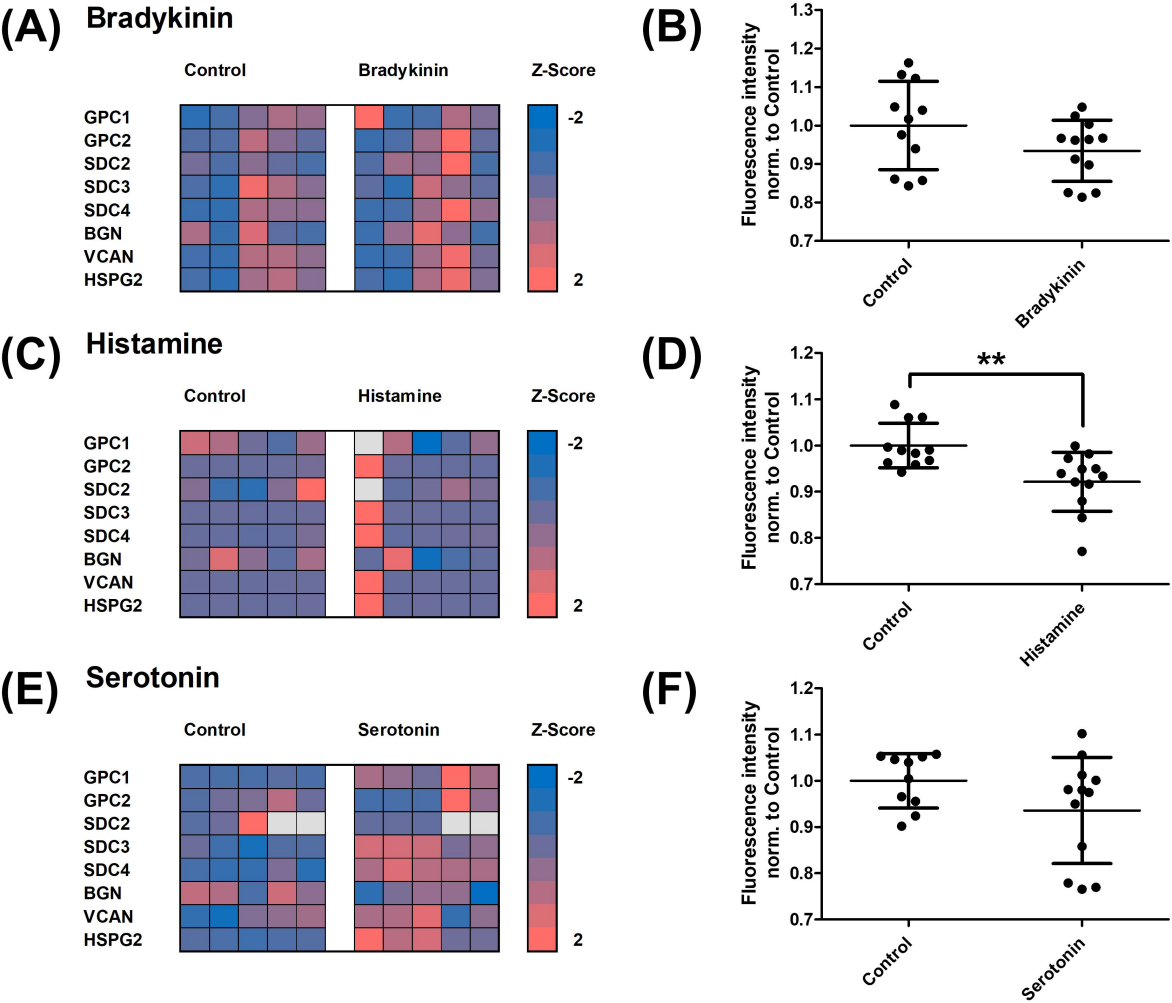
Influence of bradykinin, histamine, and serotonin on the endothelial glycocalyx. Human umbilical vein endothelial cells were treated with the respective hormone, and its impact on the glycocalyx was investigated. Bradykinin showed potentially modulated glycoproteins in the screening (**A**, particularly Syndecan 2), but these changes were not confirmed in subsequent verification experiments (**Supplementary Data 2**). No significant effect was observed in the Wheat Germ Agglutinin (WGA) assay **(B)**. Histamine, on the other hand, showed no significant influence on glycoprotein expression in the RT-PCR screening **(C)**. However, histamine caused a significant reduction in the fluorescence signal in the WGA assay **(D)**. For serotonin, potentially modulated glycoproteins were identified in the RT-PCR screening (E, particularly Glypican 1, Syndecan 3 and 4, Versican, and Perlecan), but these changes could not be confirmed in subsequent verification experiments. Serotonin also had no significant effect on the WGA signal **(F)**. Z-scores for expression changes were calculated and showed as heat map. Grey fields represent no detected expression. The other values are shown as scattered plots, and the mean value with the standard deviation is given. Each data point represents one well. Significances were calculated using the two-tailed Mann Whitney test (**p < 0.01, SDC, Syndecan; GPC, Glypican; BGN, Biglycan; VCAN, Versican; HSPG2, Perlecan).

In the next step, the impact of the angioedema-relevant hormones bradykinin, histamine, and serotonin on the endothelial glycocalyx was investigated (**Figure 4**). RT-PCR analysis of relevant expressed glycoproteins revealed significant gene changes only for treatment with bradykinin (A) and serotonin (E) in the screening. However, subsequent verification experiments using RT-PCR and Western blot showed no significant changes (**Supplementary Data 2**). For treatment with histamine, no relevant differences in glycoprotein expression were observed in the screening (C). However, histamine was the only hormone that led to a significant reduction in the WGA signal (B, D, F).

### Bradykinin and histamine modulate hyaluronidase activity

**Figure 5:**
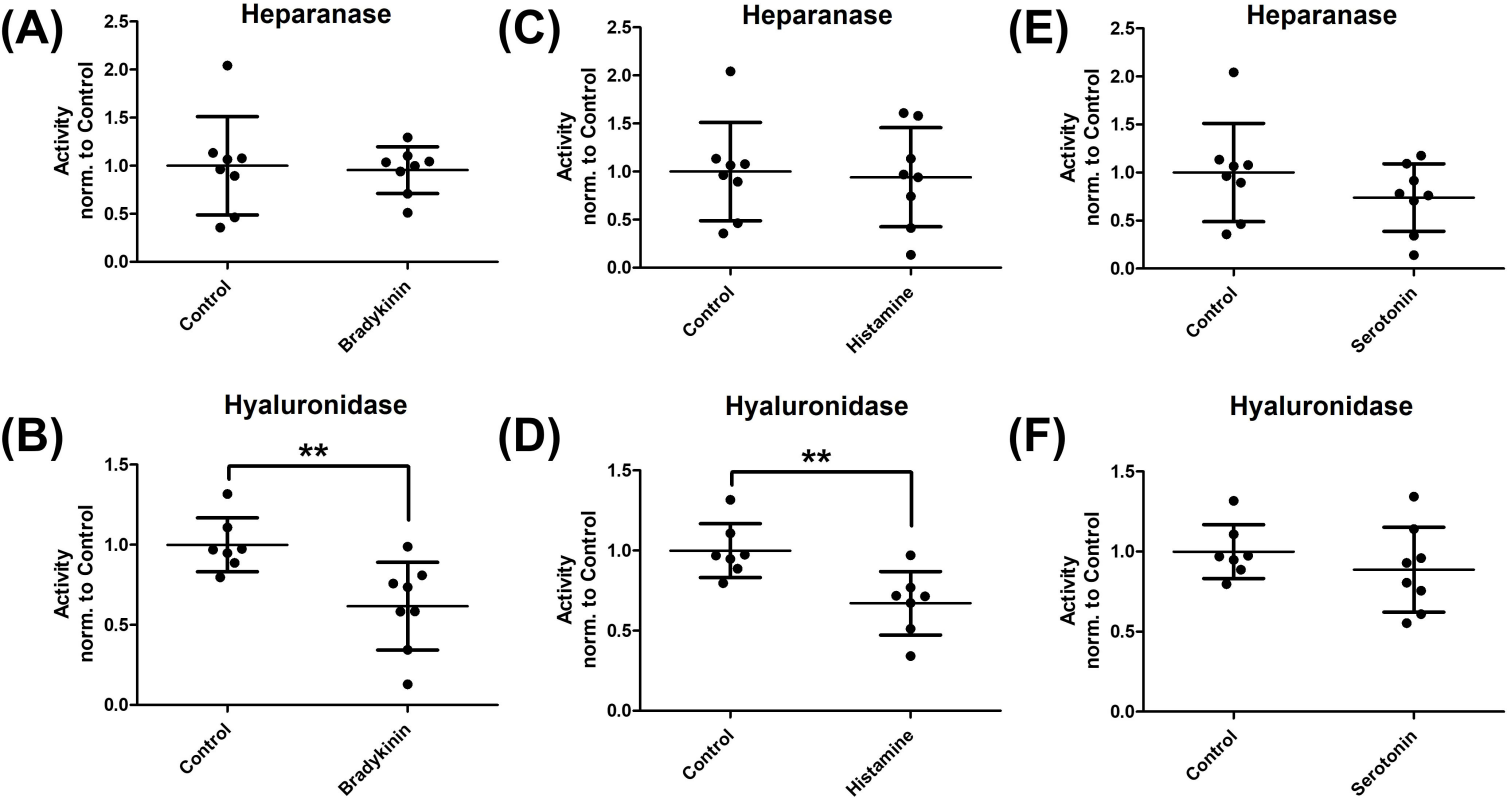
Influence of bradykinin, histamine, and serotonin on sheddase activity. To investigate the effect of the three hormones on sheddase activity, human umbilical vein endothelial cells were cultured with the respective hormones. The culture supernatant was then collected and analyzed for heparanase **(A, C, E)** and hyaluronidase **(B, D, F)** activity. While serotonin (E + F) had no effect on either sheddase, bradykinin (A + B) and histamine (C + D) led to a significant reduction in hyaluronidase activity. The values are shown as scattered plots, and the mean value with the standard deviation is given. Each data point represents one well. Significances were calculated using the two-tailed Mann Whitney test (**p < 0.01).

Next, the effect of the three hormones on the activity of glycocalyx-degrading sheddases was examined (**Figure 5**). The activity of the two main representatives - heparanase and hyaluronidase - was determined using ELISA. No relevant modulation of heparanase activity was observed A, C, E). In contrast, the addition of bradykinin and histamine led to a significant reduction in hyaluronidase activity (B, D, F).

### The endothelial glycocalyx exerts a protective effect regarding hormone-induced increased water permeability

**Figure 6:**
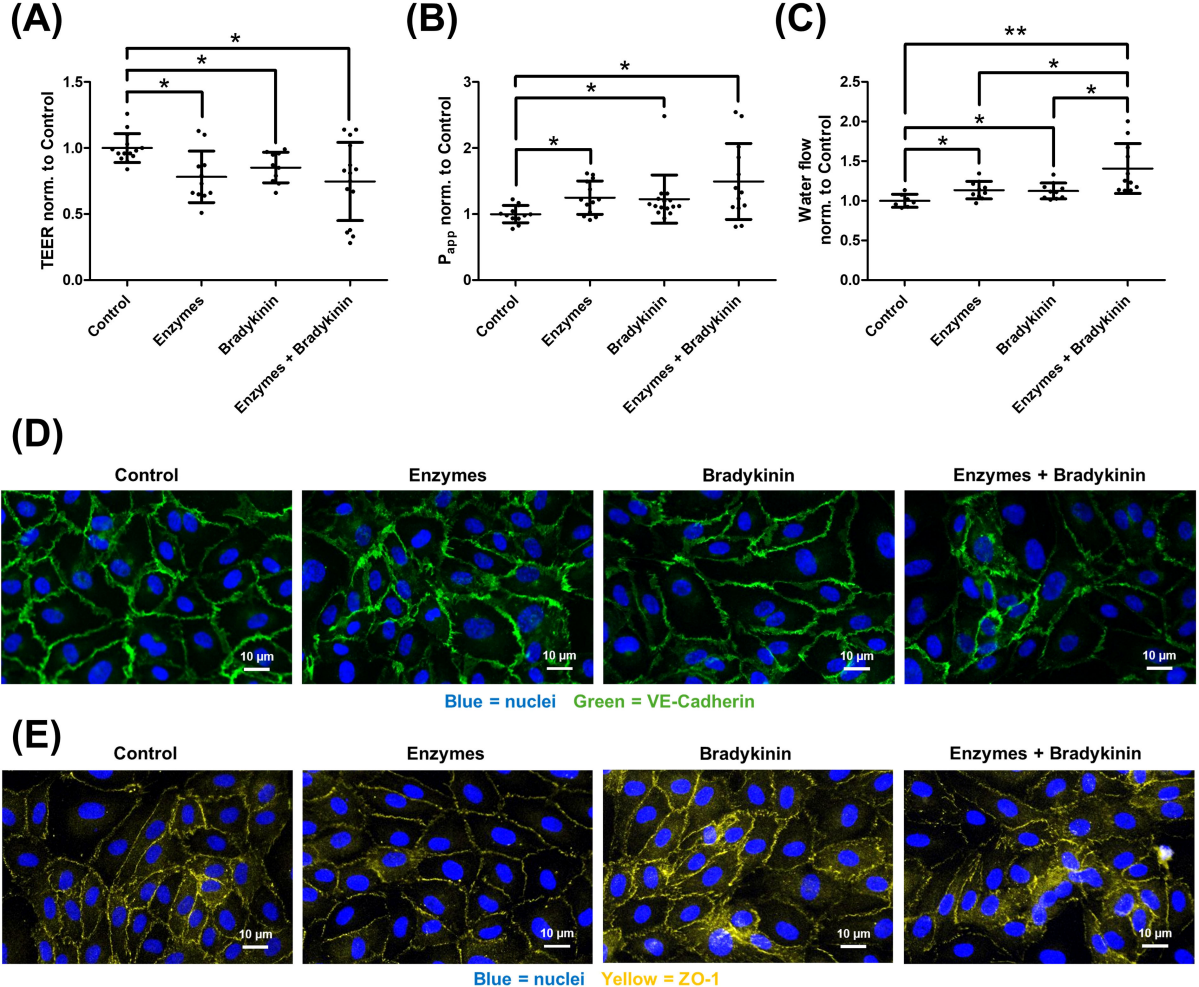
Influence of the endothelial glycocalyx on bradykinin-mediated barrier disruption. Human umbilical vein endothelial cells were cultured under four conditions: control, after enzymatic degradation of the glycocalyx (Enzymes), after bradykinin addition (Bradykinin), and a group where the glycocalyx was first degraded and then bradykinin was added (Enzymes + Bradykinin). Measurements of transendothelial electrical resistance [TEER, **(A)**] showed a decrease following glycocalyx degradation or bradykinin addition. However, the combination of both led to a decrease in TEER, but not significantly stronger than the individual components. Similar results were observed for the measurement of the apparent permeability coefficient (P_app_) for 70 kDa dextran **(B)**. Both individual components and their combination increased permeability. However, no additive effect was observed. In contrast, the determination of transendothelial water flux using the D_2_O dilution method **(C)** showed that both bradykinin and enzymatic degradation significantly increased water flux alone, but the combination of both led to a further significant increase in water flux. Corroborating these findings, immunocytochemical staining revealed alterations in cell–cell junctions. Staining for VE-cadherin [**(D)** green, nuclei in blue] and ZO-1 [**(E)** yellow, nuclei in blue] showed that enzymatic degradation alone did not markedly affect junctional localization. In contrast, bradykinin treatment resulted in a disrupted appearance of both VE-cadherin and ZO-1. A similar pattern was observed in cells where the glycocalyx was first degraded and bradykinin was subsequently applied. The values are shown as scattered plots, and the mean value with the standard deviation is given. Each data point represents one filter. Significances were calculated using the one-tailed Mann Whitney test, in case of multiple testing a Bonferroni correction was additionally performed (*p < 0.05, **p < 0.01).

After characterizing the influence of hormones on the endothelial glycocalyx, the next step was to analyze the effect of the glycocalyx on hormone-mediated barrier disruption. Four groups were formed: a control group, a group where the glycocalyx was enzymatically degraded (Enzyme), a group incubated with the respective hormone, and a group where the glycocalyx was first enzymatically degraded and then incubated with the hormone. The analysis began with bradykinin (**Figure 6**). TEER analysis showed that both glycocalyx degradation alone and bradykinin addition led to a decrease in TEER (A). However, the combination of both did not lead to an additive effect. Consistent results were observed in the P_app_ analysis (B), where it was increased by glycocalyx degradation and bradykinin addition, but no additive effect was detected. In contrast, transendothelial water flux showed different results (C). It was increased by both enzyme treatment and bradykinin addition. The combination of both, however, led to a significant further increase. Immunocytochemical staining of VE-cadherin (adherens junctions, D) and ZO-1 (tight junctions, E) showed that enzymatic glycocalyx degradation alone did not alter their localization. Bradykinin treatment resulted in a more diffuse distribution of both proteins, and the combined application of enzymes and afterwards bradykinin produced a more pronounced diffuse pattern.

**Figure 7:**
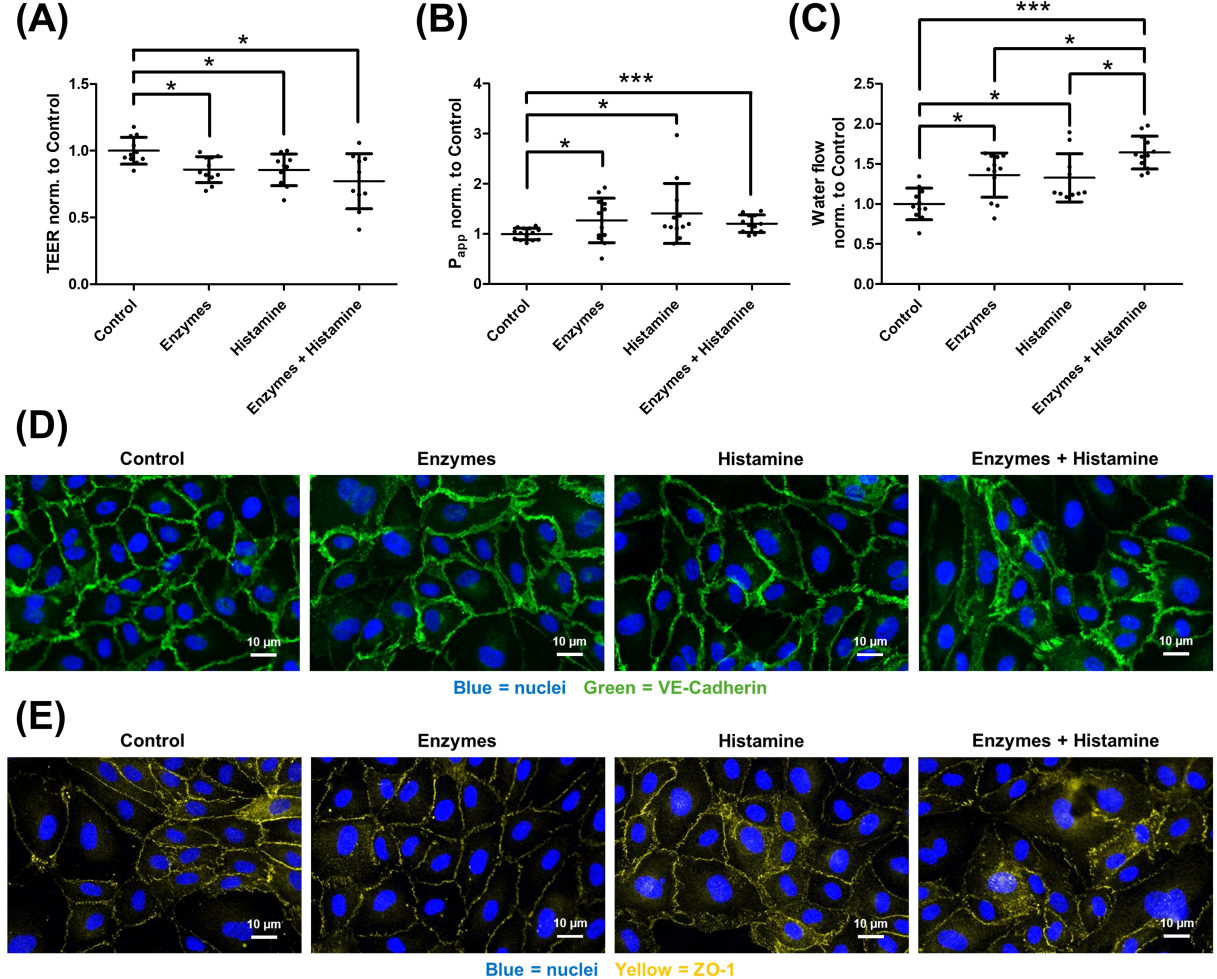
Glycocalyx is protective against histamine-induced increased transendothelial water flux. Human umbilical vein endothelial cells were cultured under four different conditions: control, after enzymatic degradation of the glycocalyx (Enzymes), after histamine treatment (Histamine), and a group where the glycocalyx was first degraded and then treated with histamine (Enzymes + Histamine). TEER measurements **(A)** revealed a decrease following either glycocalyx degradation or histamine treatment. However, the combination of both did not result in a significantly greater decrease compared to the individual treatments. Similar findings were observed when measuring the apparent permeability coefficient (P_app_) for 70 kDa dextran **(B)**, where both individual treatments and the combination increased permeability, but no additive effect was seen. In contrast, the transendothelial water flux, measured by the D_2_O dilution method **(C)**, showed that both histamine and glycocalyx degradation independently caused a significant increase in water flux. Here, the combination of both treatments resulted in a further significant increase in water flux. Immunocytochemical staining revealed corresponding changes in cell–cell junctions. VE-cadherin [**(D)** green, nuclei in blue] and ZO-1 [**(E)** yellow, nuclei in blue], were largely unaffected by enzymes alone. Histamine induced a fragmented junctional pattern, which was also observed when glycocalyx degradation preceded histamine. The values are shown as scattered plots, and the mean value with the standard deviation is given. Each data point represents one filter. Significances were calculated using the one-tailed Mann Whitney test, in case of multiple testing a Bonferroni correction was additionally performed (*p < 0.05, ***p < 0.001).

Similar findings were observed for histamine (**Figure 7**). Here, enzymatic degradation of the glycocalyx and addition of histamine also led to a decrease in TEER (A) and an increase in P_app_ (B) and water flux (C). As with bradykinin, no additive effect was observed for TEER and P_app_, but a significant increase in water flux was seen with the combination of both. Staining for VE-cadherin (D) and ZO-1 (E) demonstrated no detectable changes after enzymatic glycocalyx degradation alone. Histamine treatment led to a broader and less defined localization of both proteins, with a more accentuated diffuse appearance when glycocalyx was degraded before histamine application.

**Figure 8:**
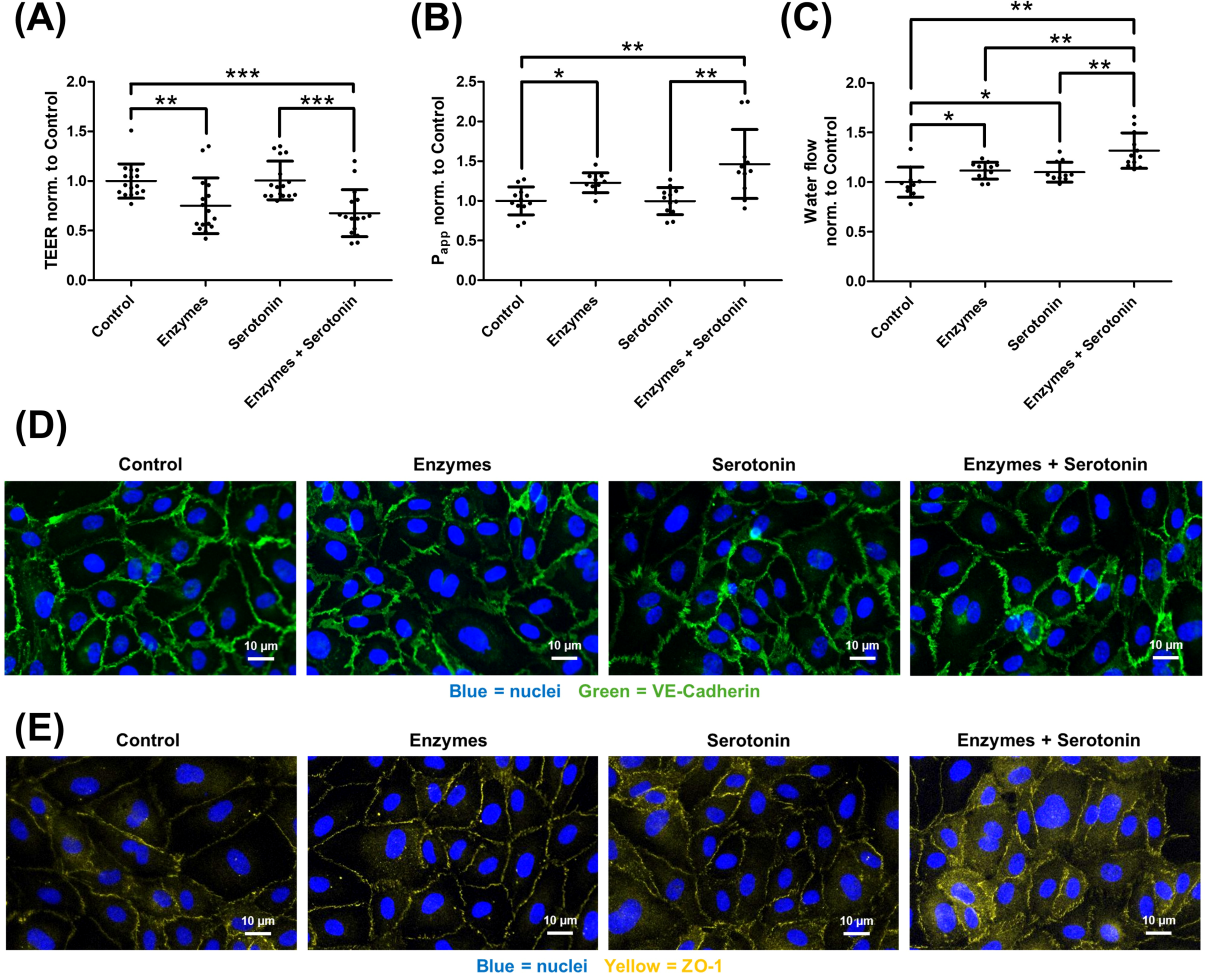
Serotonin increases transendothelial water flux, with the glycocalyx counteracting this effect. Human umbilical vein endothelial cells were cultured under four conditions. Control, after enzymatic degradation of the glycocalyx (Enzymes), after serotonin treatment (Serotonin), and a group where the glycocalyx was first degraded and then treated with serotonin (Enzymes + Serotonin). Serotonin alone had no effect on the transendothelial electrical resistance [TEER, **(A)**], unlike the degradation of the endothelial glycocalyx. The combination of both treatments did not result in a significant enhancement of the effect. For the measurement of the apparent permeability coefficient (P_app_) for 70 kDa dextran **(B)**, no effect of serotonin was observed. However, glycocalyx degradation increased permeability, but no additive effect was seen. In contrast, the D_2_O dilution method revealed that serotonin significantly increased transendothelial water flux, as did glycocalyx degradation **(C)**. Unlike TEER and P_app_, the combination of both treatments resulted in a significant increase in the effect. Immunocytochemical staining showed mild junctional alterations in response to serotonin, primarily affecting ZO-1 [**(E)** yellow, nuclei in blue], while VE-cadherin [**(D)** green, nuclei in blue] remained largely unchanged. The values are shown as scattered plots, and the mean value with the standard deviation is given. Each data point represents one filter. Significances were calculated using the one-tailed Mann Whitney test, in case of multiple testing a Bonferroni correction was additionally performed (*p < 0.05, **p < 0.01, ***p < 0.001).

Regarding serotonin, slightly different but still consistent observations were made (**Figure 8**). The addition of the enzymes decreased TEER (A) and increased P_app_ (B) and water flux (C). Serotonin, however, had no significant effect on TEER and P_app_. Though, a significant increase in transendothelial water flux was observed with serotonin. Similar to bradykinin and histamine, no additive effect was seen for TEER and P_app_, but the combination of both resulted in a significant enhancement of the water flux effect. For VE-cadherin (D) and ZO-1 (E), enzymatic glycocalyx degradation alone showed no change in staining patterns. Serotonin treatment did not appreciably affect VE-cadherin localization, while ZO-1 appeared slightly more diffuse; this effect was modestly enhanced when cells were pretreated with enzymes before serotonin application.

## Discussion

This is the first study to examine the impact of structures of the glycocalyx on angioedema development. The existence of a glycocalyx on HUVEC was demonstrated using the WGA assay. This assay was also utilized by Abe et al. to detect GAGs, where they observed a significantly reduced fluorescence intensity of FITC-conjugated WGA after enzymatic degradation of the glycocalyx by hyaluronidase, heparanase, and neuraminidase (30). Similarly, in this study, fluorescence intensity significantly decreased after degradation by each enzyme (**Figures 1A, B**). WGA predominantly binds to sialic acids, which are specifically cleaved from glycans by neuraminidase. The other enzymes reduce the fluorescence signal by degrading different GAGs or proteoglycans, such as syndecan-1, which has terminally attached sialic acids (31, 32). A methodological limitation of this study concerns the structural assessment of proteoglycan degradation following enzymatic glycocalyx removal. Western blot and ELISA are of limited suitability for evaluating proteoglycan integrity, as these highly heterogeneous macromolecules with complex GAG side chains are not reliably resolved by standard SDS-PAGE without prior removal of GAGs, which results in loss of information about their native structural state. Since the enzymes used in this study selectively cleave GAG side chains without degrading the corresponding core proteins, immunodetection of core proteins would not accurately reflect enzymatic efficiency. Moreover, ELISA-based approaches do not provide information about the structural integrity of the complete proteoglycan molecule, and neoepitope-specific antibodies are not broadly available (15, 33, 34). Therefore, we relied on WGA binding as a global measure of glycocalyx integrity and complemented this with functional permeability analyses. Without enzymatic degradation, the glycocalyx structure remains relatively stable in the WGA assay over a period of four days (**Figure 1C**). The expression pattern of the glycocalyx over seven days shows an increase in syndecan-4 and biglycan in RT-PCR analysis (**Figure 1D**). Liu et al. demonstrated an upregulation of syndecan-1 and -4 gene expression in HUVEC after application of shear stress (35). This indicates that these two syndecans play a key role in cytoskeletal adaptation to external forces such as shear stress and are also increasingly expressed during cell proliferation (14, 36). Biglycan gene expression appears to correlate with cell proliferation as well, given its previously described anti-apoptotic effect via membrane stabilization and its upregulation in colorectal carcinoma, promoting tumor cell migration and proliferation (37, 38). Other genes show a decline in expression on day four, with most returning to initial levels by day seven. Light and fluorescence microscopy also revealed maximum confluency on day four, indicating that cell proliferation and gene expression are temporarily reduced. By day seven of cell culture, increased synthesis could be triggered by enzyme-induced glycocalyx degradation, leading to a steady-state equilibrium of glycocalyx synthesis and degradation. This is supported by the increasing heparanase activity on day four (**Figure 1E**). In contrast, hyaluronidase activity decreases on day two and does not fully recover by day four. Hyaluronidase is known to have a very short half-life of approximately three minutes, suggesting that its resynthesis and activity might also decrease due to a temporarily reduced synthesis rate in endothelial cells (39). These results demonstrate that HUVEC represent a suitable and reproducible model to investigate structural and functional aspects of the endothelial glycocalyx. However, several limitations of the experimental system should be considered. The HUVEC monolayer model does not fully recapitulate the *in vivo* vascular microenvironment, including shear stress–dependent remodeling, three-dimensional vessel architecture, and interactions with circulating inflammatory cells. These factors may influence glycocalyx structure and endothelial responsiveness under physiological conditions. Therefore, *in vivo* validation will be required to determine the extent to which our findings translate into intact vascular systems, although the controlled *in vitro* approach enables precise mechanistic analysis.

The glycocalyx constitutes a crucial component of the endothelial barrier. Its degradation leads to a decrease in TEER and an increase in permeability for macromolecules and water (**Figures 2A–C**). Similar effects on TEER have been reported in other studies (40–42). Gao and Lipowsky observed increased permeability to 70 kDa dextrans after application of chondroitinase and hyaluronidase, whereas heparanase had the opposite effect. They proposed that glycocalyx collapse due to heparan sulfate degradation results in an increased matrix density, thereby maintaining filtration stability. Importantly, however, Gao and Lipowsky assessed diffusion properties within the glycocalyx layer itself in intact perfused microvessels, determining diffusion coefficients of macromolecules within the glycocalyx matrix. In contrast, our study evaluates permeability across the entire endothelial monolayer, including intercellular junctions and transcellular pathways. Thus, the differing effects observed after heparanase treatment likely reflect distinct levels of barrier assessment—matrix-internal diffusion versus transendothelial permeability—rather than fundamentally opposing biological mechanisms (43). They proposed that glycocalyx collapse due to heparan sulfate degradation results in an increased density, maintaining filtration stability. Delgadillo et al. found a TEER increase after enzymatic glycocalyx degradation by heparanase and hyaluronidase but did not observe a significant increase in permeability for 10 kDa dextrans (42). This might be explained by the presence of physiological glycocalyx pores of up to 7 nm in diameter, allowing smaller molecules like 10 kDa dextrans to diffuse regardless of glycocalyx integrity (21). In contrast, the 70 kDa dextrans used in this study, with a diameter of approximately 10.90 nm, can only pass through a compromised glycocalyx. 70 kDa corresponds to the size of the plasma protein albumin, which plays a key role in maintaining colloid osmotic pressure, thereby retaining water within the intravascular space (44). Consequently, increased permeability for similarly sized particles may play a significant role in the development of edema. Besides increased permeability to particles, water flow is particularly relevant in the development of angioedema. Using the D_2_O dilution method (29), an increased water flow was detected after glycocalyx degradation for all enzymes except chondroitinase under a hydrostatic pressure gradient (**Figure 2C**). In addition to its barrier-enhancing effect, the glycocalyx also has water-binding properties, with hyaluronic acid playing a key role by forming a three-dimensional network through hydrogen bonds (45). When this network is degraded by hyaluronidase, water retention within the glycocalyx is impaired, allowing water and other molecules to pass through the endothelium. Chondroitin sulfate appears to have minimal impact on water flux in these experiments, likely due to its relatively low abundance of approximately 20% of the GAGs within the glycocalyx, rendering its degradation insignificant (46). It is well established that paracellular transport is ion-specific and regulated by tight junctions. Claudin-4 and -11 reduce cation conductivity, whereas Claudin-2 and -15 increase it (47). Interestingly, enzymatic glycocalyx degradation did not significantly alter ion selectivity. In our ion substitution experiments (**Figure 2D**), a slightly higher TEER was observed in sodium aspartate buffer compared to chloride-lysine buffer, indicating a greater restriction of cation conductivity than anion conductivity. This finding may reflect the sodium-binding capacity of the negatively charged GAG side chains within the glycocalyx (48). However, a comparable pattern persisted after enzymatic glycocalyx degradation, suggesting that overall ion selectivity of the endothelial barrier is predominantly governed by tight junction proteins or ion channels rather than the apical glycocalyx layer. Given that small ions such as Na^+^ and Cl^−^ are several orders of magnitude smaller than the estimated pore size of the glycocalyx, they can readily diffuse through this structure, whereas paracellular charge selectivity is mainly determined by claudins and other tight junction components (49). Consistent with this concept, glycocalyx degradation alone did not markedly affect junctional organization in our immunocytochemical analyses, supporting the conclusion that ion selectivity is regulated primarily at the level of intercellular junctions, while the glycocalyx predominantly modulates macromolecular and water permeability.

Importantly, the observed changes in macromolecular and water permeability are not due to apoptotic effects of glycocalyx degradation on the endothelial cells, as confirmed by both metabolic and microscopic analyses (**Figures 3A–D**). Therefore, these effects appear to reflect a modulation of endothelial barrier function rather than cell viability loss.

The composition of the glycocalyx does not appear to be significantly altered by the mediators bradykinin and serotonin, as neither consistent modulation of glycoprotein gene expression nor degradation of glycocalyx components were detected in the WGA assay (**Figures 4A, B, E, F**; **Supplementary Figures 2A–H**). Although the initial RT-PCR screening suggested modest alterations in selected glypican transcripts, these changes were small and could not be reproduced in subsequent validation experiments. Moreover, no corresponding changes were observed at the protein level or in WGA-based glycocalyx integrity measurements. Taken together, these findings indicate that the observed transcriptional fluctuations likely reflect minor and biologically non-robust effects rather than relevant structural modulation of the endothelial glycocalyx under the conditions studied. Van Teeffelen et al. hypothesized that nitric oxide (NO) released by bradykinin may alter glycocalyx charge through interactions with its components, leading to structural changes (50). However, potential polysaccharide restructuring cannot be detected in the WGA assay. Histamine, on the other hand, led to a reduction in fluorescence signal in the WGA assay, indicating glycocalyx damage (**Figure 4D**). This may be indirectly caused by reactive oxygen species, which are increasingly produced following upregulation of endothelial nitric oxide synthase by histamine (51). Enzyme activity assays did not show heparanase activation by bradykinin, histamine, or serotonin (**Figures 5A, C, E**). However, hyaluronidase activity significantly decreased with bradykinin and histamine (**Figures 5B, D**). The weakening of the endothelial barrier by these mediators may be compensated by endothelial inhibition of hyaluronidase, preserving high-molecular-weight hyaluronic acid, which has anti-inflammatory and immune-supportive effects (52). TEER and apparent permeability coefficients confirm endothelial barrier impairment by histamine and bradykinin, though no additive effect was observed following enzymatic glycocalyx degradation (**Figures 6A, B**, **7A, B**). The concentrations of bradykinin (100 µM), histamine (0.1 µM), and serotonin (100 µM) were selected based on prior concentration–response analyses (**Supplementary Data 3**) and are consistent with concentrations previously shown to induce changes in transendothelial water permeability using the established D_2_O dilution method (29). The permeability-enhancing effect of histamine likely occurs through modulation of cell-cell contacts, activating various signalling pathways via the H1 receptor, including VE-cadherin phosphorylation (53, 54). Bradykinin compromises the endothelial barrier by disrupting tight junctions through VE-cadherin and Claudin-5 downregulation (55). Thus, histamine, bradykinin, and serotonin do not seem to directly impact the glycocalyx.

Following enzymatic degradation of the glycocalyx, all mediators promoted an increase in transendothelial water permeability (**Figures 6C**, **7C**, **8C**). This effect may be explained by enhanced accessibility of mediator receptors once the glycocalyx barrier is compromised. In addition, weakening of the glycocalyx amplifies mediator-induced alterations in cell–cell junctions, thereby further elevating endothelial permeability. Consequently, the glycocalyx exerts a protective role in preventing excessive water flux in response to angioedema-relevant mediators such as bradykinin, histamine, and serotonin. Immunocytochemical staining revealed that both bradykinin and histamine modulate VE-cadherin, a key protein of adherens junctions, and ZO-1, a critical tight junction protein, leading to a more diffuse localization (**Figures 5D, E**, **7D, E**). In contrast, the effect of serotonin was weaker overall and largely restricted to ZO-1 (**Figures 8D, E**). These observations are consistent with the results obtained from TEER, P_app_, and water permeability measurements (**Figures 8A–C**). Glycocalyx degradation alone had no notable impact. However, in cells pretreated with glycocalyx-degrading enzymes followed by mediator addition, the effect of the mediators was slightly enhanced, supporting the hypothesis that mediator access to their receptors is improved, thereby amplifying their action. For precise mechanistic conclusions, further studies are required. Until now, such direct analyses of transendothelial water permeability were not feasible. The D_2_O dilution technique, for the first time, enables quantitative assessment of how bradykinin, histamine, serotonin, and the integrity of the glycocalyx influence water flux across the endothelium. This additional readout is particularly important because the protective contribution of the glycocalyx would not have been identifiable through TEER or P_app_ measurements alone. In the context of elucidating the mechanistic basis of angioedema formation, the ability to directly quantify water permeability therefore represents a significant methodological advancement. Beyond this functional sensitization, additional mechanisms may contribute to the protective role of the glycocalyx. The protective role of the glycocalyx observed in our study is likely multifactorial and extends beyond a purely physical barrier function. In addition to steric and electrostatic filtering at the endothelial surface, several mechanisms support this concept. First, glycocalyx degradation has been shown to initiate intracellular signaling processes that modulate endothelial behavior, indicating an active regulatory role rather than passive structural loss (30). Second, shedding of glycocalyx components may generate bioactive fragments capable of directly impairing endothelial barrier integrity (56). Third, the glycocalyx together with the underlying actin-rich cortex forms a dynamic nanomechanical unit that regulates barrier stability and responds to inflammatory or mechanical stimuli (57, 58). These mechanisms provide a molecular framework explaining why glycocalyx disruption enhances mediator-induced barrier dysfunction beyond simple receptor shielding.

Taken together, our findings provide new insight into the role of the endothelial glycocalyx in modulating mediator-induced barrier dysfunction in angioedema. Nevertheless, several limitations and future perspectives should be considered. The HUVEC monolayer model does not fully reproduce the *in vivo* vascular microenvironment, including shear stress, multicellular interactions, and vascular bed–specific heterogeneity. Future studies employing flow-based systems, organ-on-chip approaches, or *in vivo* models will be required to validate these findings under more physiological conditions. Moreover, investigation of endothelial cells derived from patients with different angioedema subtypes may help to identify disease-specific alterations of glycocalyx composition and barrier responsiveness. Such approaches could further bridge the gap between mechanistic *in vitro* observations and clinical relevance.

In addition to mechanistic refinement in future experimental models, the translational relevance of glycocalyx alterations merits further exploration. Given the central role of the glycocalyx in regulating endothelial permeability and mediator responsiveness, glycocalyx-derived components may represent promising biomarkers for angioedema. Mechanical stress and immune responses are both known triggers of angioedema (59). In the context of such stimuli, the endothelial glycocalyx may undergo structural degradation. This process, referred to as shedding, involves the detachment of glycocalyx components from the endothelial surface. As a result, the integrity of the glycocalyx is reduced, increasing risk for angioedema, and its fragments become detectable in circulation. Shedding products such as hyaluronan, heparan sulfate, and syndecan ectodomains are detectable in plasma and reflect structural alterations of the endothelial surface layer (60). In particular, Syndecan-1 and heparan sulfate have emerged as clinically relevant indicators of vascular injury across a broad spectrum of inflammatory and critical illnesses, with reported associations to disease severity and outcomes (25, 61). In the specific context of angioedema, elevated endothelial and vascular integrity markers have recently been demonstrated in different disease subtypes, underscoring the contribution of endothelial dysfunction to pathophysiology (62). Circulating glycocalyx components may therefore complement established endothelial biomarkers by directly reflecting remodeling of the endothelial surface layer that accompanies permeability disturbances. Interestingly, the recently described form of hereditary angioedema caused by mutations in *HS3ST6*, encoding heparan sulfate 3-O-sulfotransferase 6, further supports a potential pathophysiological link between heparan sulfate metabolism and bradykinin-mediated angioedema (63). This observation suggests that alterations in endothelial surface glycosaminoglycans may not only modulate barrier function but could also influence kallikrein-kinin-system activation or mediator responsiveness. Thus, the endothelial glycocalyx may represent a clinically relevant interface in specific genetic forms of angioedema. However, specificity remains a challenge, as glycocalyx shedding also occurs in other inflammatory and vascular disorders. Furthermore, the dynamic turnover of glycocalyx components and potential vascular bed–specific differences require careful temporal and clinical interpretation. Nevertheless, in combination with established endothelial markers and functional assessments of barrier integrity, glycocalyx-associated biomarkers hold translational potential for improving mechanistic understanding, disease stratification, and monitoring of angioedema.

## Data Availability

The original contributions presented in the study are included in the article/**Supplementary Material**. Further inquiries can be directed to the corresponding author.

## Supplementary material

Supplementary Figure 1 Screening of glycoproteins expressed in human endothelial cells. Human umbilical vein endothelial cells (HUVEC) were cultured for four days, and the expression of selected glycoproteins was quantified by real-time polymerase chain reaction **(A)**. A threshold for relevant expression was defined as ≥0.0001 relative to GAPDH. The values are shown as scattered plots, and the mean value with the standard error of the mean is given. Each data point represents one well (GAPDH, Glycerinaldehyd-3-phosphat-dehydrogenase; GPC, Glypican; SDC, Syndecan; OGN, Osteoglycin; BGN, Biglycan; VCAN, Versican; HSPG2, Perlecan).

Supplementary Figure 2 Verification of missing bradykinin- and serotonin-induced effects on glycoprotein expression. To validate the effects of bradykinin and serotonin observed in **Figure 4** independent batches of human umbilical vein endothelial cells (HUVEC) were treated with the respective hormone. Bradykinin-induced downregulation of SDC2 was confirmed at the mRNA level **(A)**, but this effect was not detectable at the protein level by western blot analysis **(B)**. For serotonin, the previously observed effects on SDC3 **(E)**, SDC4 **(F)**, VCAN **(G)**, and HSPG2 **(H)** could not be reproduced in the validation experiments. In contrast, serotonin-mediated downregulation of GPC1 at the mRNA level was confirmed **(C)**, although this finding was not supported by western blot analysis **(D)**. The values are shown as scattered plots, and the mean value with the standard deviation is given. Each data point represents one well. Significances were calculated using the two-tailed Mann Whitney test (* = p < 0.05, ** = p < 0.01, GAPDH, Glycerinaldehyd-3-phosphat-dehydrogenase; SDC, Syndecan; GPC, Glypican; BGN, Biglycan; VCAN, Versican; HSPG2, Perlecan).

Supplementary Figure 3 Concentration–response analysis of bradykinin, histamine, and serotonin on endothelial barrier function. Transendothelial electrical resistance (TEER; **A–C**) and apparent permeability (P_app_; **D–F**) were assessed in human umbilical vein endothelial cell (HUVEC) monolayers after stimulation with increasing concentrations of bradykinin **(A, D)**, histamine **(B, E)**, and serotonin **(C, F)**. Concentrations are shown as log_10_-transformed values [µM]. TEER values are expressed in Ω·cm², and P_app_ values are normalized to untreated control conditions. Data points represent mean ± SEM. Nonlinear regression curves (red lines) illustrate the concentration–response relationship. These analyses served as the basis for selecting the concentrations used in subsequent functional experiments.
