# Supplementary figures and images for: Glycocalyx strengthens endothelial barrier function and protects from angioedema inducing compounds
Source: Front Immunol. 2026 Mar 12;17:1758997. doi: 10.3389/fimmu.2026.1758997 (PMC13017264; doi:10.3389/fimmu.2026.1758997)

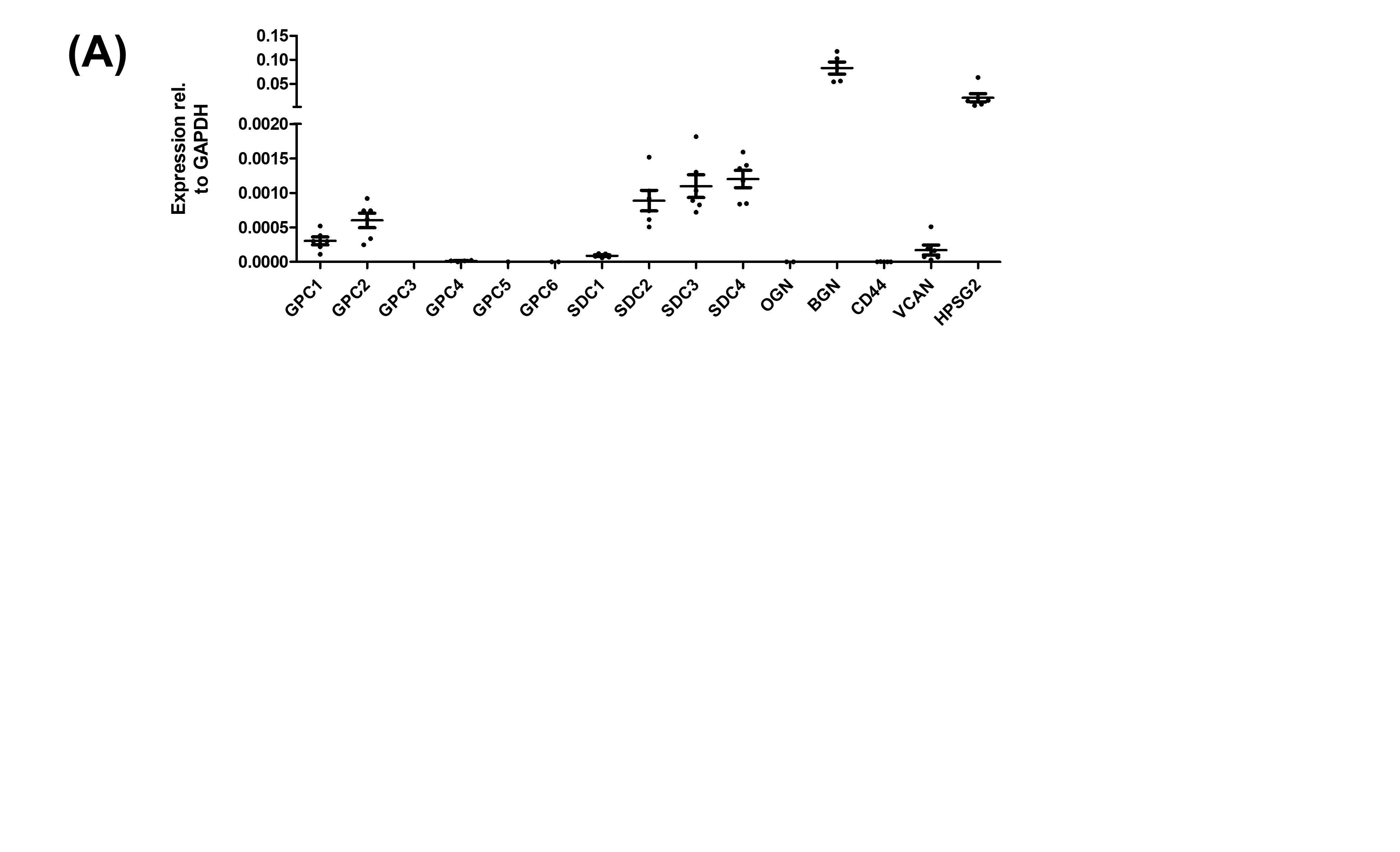

Supplement: Supplementary Figure 1 — Screening of glycoproteins expressed in human endothelial cells. Human umbilical vein endothelial cells (HUVEC) were cultured for four days, and the expression of selected glycoproteins was quantified by real-time polymerase chain reaction (A). A threshold for relevant expression was defined as ≥0.0001 relative to GAPDH. The values are shown as scattered plots, and the mean value with the standard error of the mean is given. Each data point represents one well (GAPDH, Glycerinaldehyd-3-phosphat-dehydrogenase; GPC, Glypican; SDC, Syndecan; OGN, Osteoglycin; BGN, Biglycan; VCAN, Versican; HSPG2, Perlecan). [file Image1.jpeg]

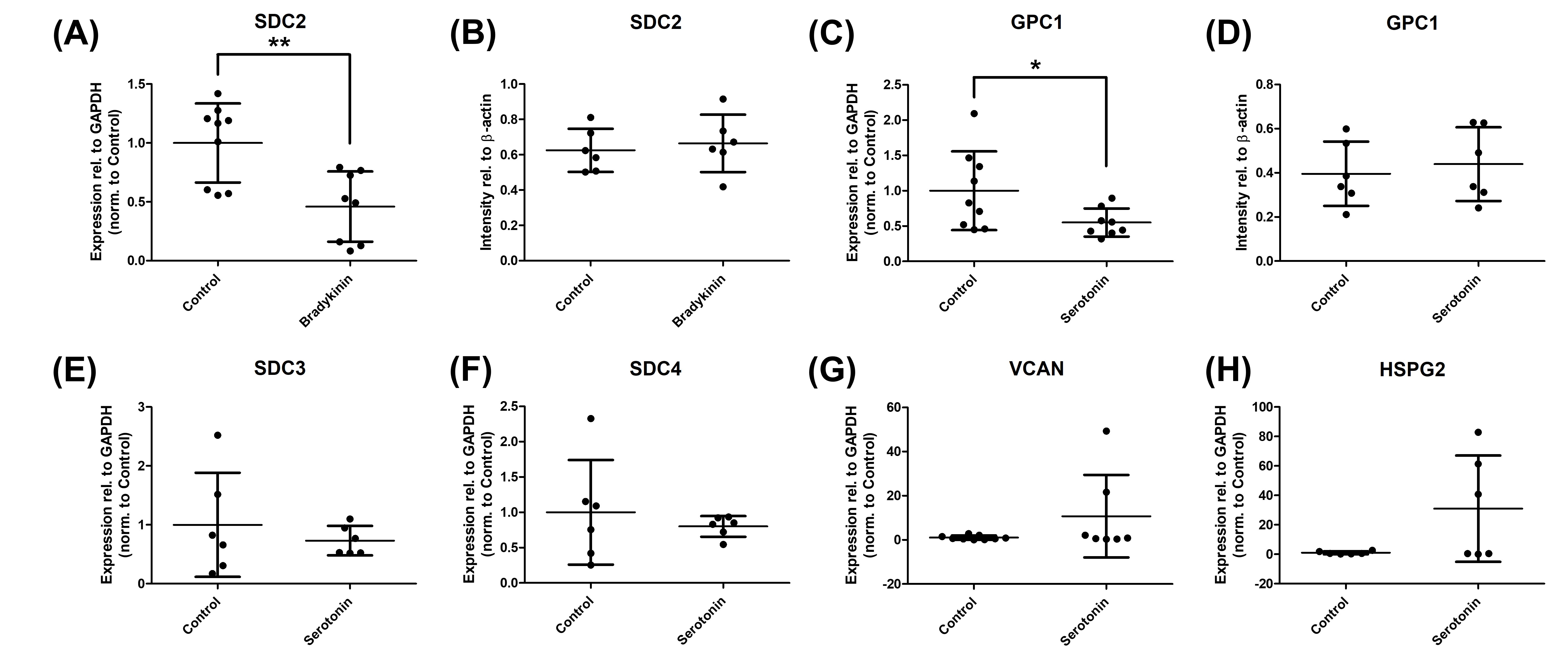

Supplement: Supplementary Figure 2 — Verification of missing bradykinin- and serotonin-induced effects on glycoprotein expression. To validate the effects of bradykinin and serotonin observed in Figure 4 independent batches of human umbilical vein endothelial cells (HUVEC) were treated with the respective hormone. Bradykinin-induced downregulation of SDC2 was confirmed at the mRNA level (A), but this effect was not detectable at the protein level by western blot analysis (B). For serotonin, the previously observed effects on SDC3 (E), SDC4 (F), VCAN (G), and HSPG2 (H) could not be reproduced in the validation experiments. In contrast, serotonin-mediated downregulation of GPC1 at the mRNA level was confirmed (C), although this finding was not supported by western blot analysis (D). The values are shown as scattered plots, and the mean value with the standard deviation is given. Each data point represents one well. Significances were calculated using the two-tailed Mann Whitney test (* = p < 0.05, ** = p < 0.01, GAPDH, Glycerinaldehyd-3-phosphat-dehydrogenase; SDC, Syndecan; GPC, Glypican; BGN, Biglycan; VCAN, Versican; HSPG2, Perlecan). [file Image2.jpeg]

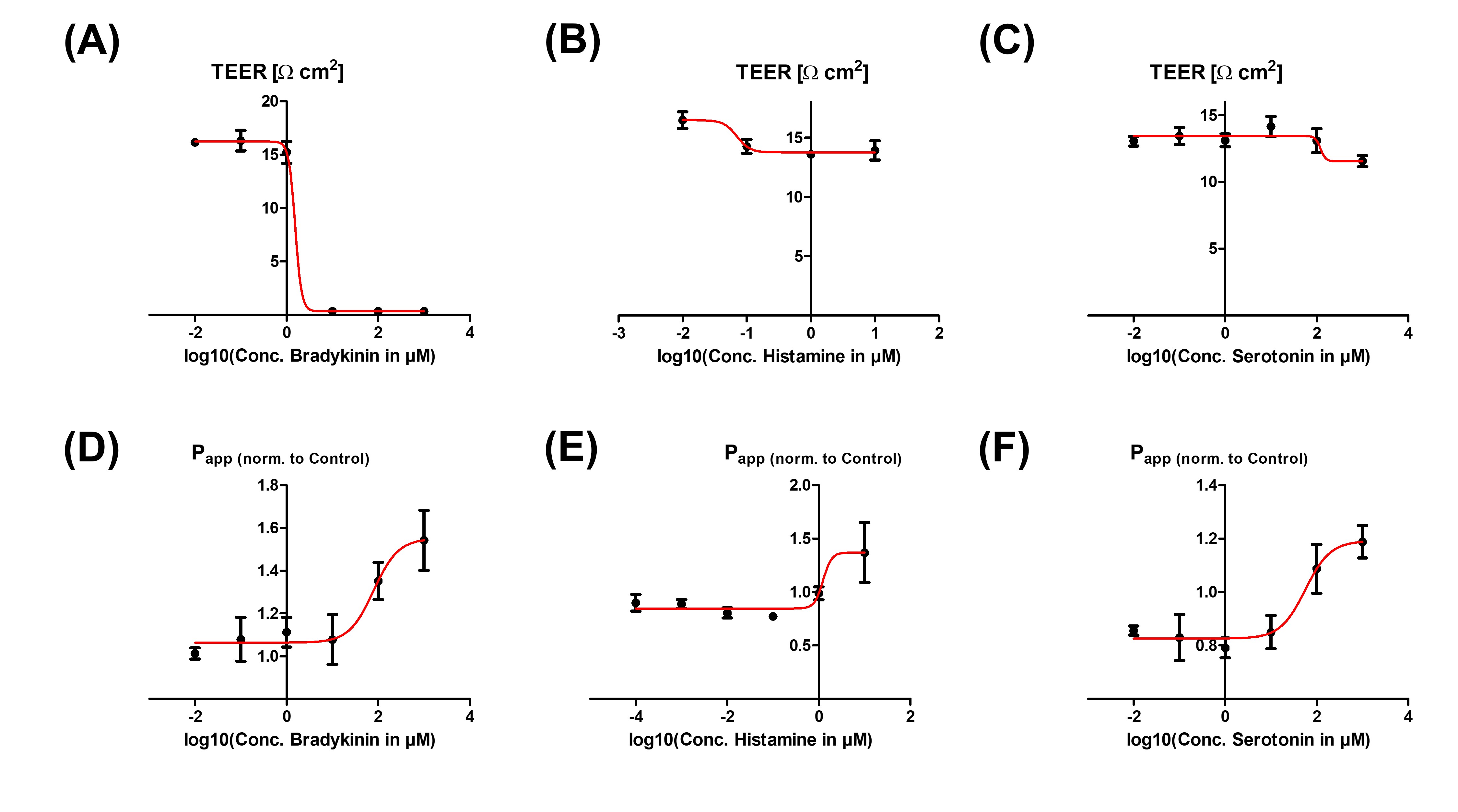

Supplement: Supplementary Figure 3 — Concentration–response analysis of bradykinin, histamine, and serotonin on endothelial barrier function. Transendothelial electrical resistance (TEER; A–C) and apparent permeability (Papp; D–F) were assessed in human umbilical vein endothelial cell (HUVEC) monolayers after stimulation with increasing concentrations of bradykinin (A, D), histamine (B, E), and serotonin (C, F). Concentrations are shown as log10-transformed values [µM]. TEER values are expressed in Ω·cm², and Papp values are normalized to untreated control conditions. Data points represent mean ± SEM. Nonlinear regression curves (red lines) illustrate the concentration–response relationship. These analyses served as the basis for selecting the concentrations used in subsequent functional experiments. [file Image3.jpeg]
